# The Impact of Salinity in the Irrigation of a Wild Underutilized Leafy Vegetable, *Sonchus oleraceus* L.

**DOI:** 10.3390/plants13111552

**Published:** 2024-06-04

**Authors:** Anna Gkotzamani, Ioannis Ipsilantis, George Menexes, Andreas Katsiotis, Konstadinos Mattas, Athanasios Koukounaras

**Affiliations:** 1 Laboratory of Vegetable Crops, School of Agriculture, Aristotle University of Thessaloniki, 54124 Thessaloniki, Greece; agkotzam@agro.auth.gr; 2 Soil Science Laboratory, School of Agriculture, Aristotle University of Thessaloniki, 54124 Thessaloniki, Greece; iipsi@agro.auth.gr; 3 Laboratory of Agronomy, School of Agriculture, Aristotle University of Thessaloniki, 54124 Thessaloniki, Greece; gmenexes@agro.auth.gr; 4 Department of Agricultural Sciences, Biotechnology and Food Science, Faculty of Geotechnical Sciences and Environmental Management, Cyprus University of Technology, 50329 Limassol, Cyprus; andreas.katsiotis@cut.ac.cy; 5 Department of Agricultural Economics, School of Agriculture, Aristotle University of Thessaloniki, 54124 Thessaloniki, Greece; mattas@auth.gr

**Keywords:** abiotic stresses, climate change, biodiversity, unexploited species, neglected crops, puha

## Abstract

Introducing non- or under-utilized crops to cultivation generates benefits such as biodiversity enrichment, supporting mitigation actions towards climate change-induced effects. The salinization of soil and water supplies is progressively disrupting natural habitats and food production, especially in regions such as the Mediterranean. *Sonchus oleraceus* L. is a Mediterranean wild leafy green with nutritional and medicinal properties. This study’s purpose was to determine whether salinity affects the growth, quality, and nutrient composition of *Sonchus oleraceus* L. In an unheated plastic greenhouse, seedlings were transplanted in pots filled with perlite and irrigated with a nutrient solution with no NaCl added (the control, C) or with the addition of 40, 60, 80, and 100 mM of NaCl (treatments S4, S6, S8, and S10, respectively). The leaf and root growth, leaf quality, and the nutrient composition of leaves and roots were determined. Regarding the results, growth was mainly affected at high salinity levels (S8 and S10), with no observed effects of salinity on the determined quality parameters. The nutrient composition was variably affected by salinity in leaves but not in roots (except in the case of Na and the K/Na ratio). *Sonchus oleraceus* L. showed a general relative tolerance in moderate salinity levels (40 and 60 mM of NaCl), suggesting potential commercial exploitation of the species in areas where the quality of irrigation water is low. However, the health effects of consuming this species grown under salinity stress need to be studied in future research.

## 1. Introduction

Salinization is an increasing problem worldwide, resulting from natural processes and human activities [1]. Intensive agriculture has led to high levels of salinity in cultivable areas and water supplies [2], and approximately 30% of the global irrigated land is considered salt-affected due to anthropogenic salinization [3]. Irrigation water is one of the most significant parameters of soil salinization. The use of saline or highly fertilized irrigation water can increase the concentration of salts in the soil solution [4]. Based on electrical conductivity (EC), water can be classified as non-saline (<0.7 mS/cm), slightly saline (0.7–2 mS/cm), moderately saline (2–10 mS/cm), highly saline (10–25 mS/cm), and very highly saline (25–45 mS/cm) [5]. Salinity interrupts the exchange of water and nutrients in the root system with the soil and affects photosynthesis [6], causing a reduction in growth and development [2]. Additionally, salinity threatens biodiversity and interrupts the ecosystem, causing desertification, diminishing the biological activity of the soil [7], and leading to a threat to global food security [8]. Plants’ susceptibility or tolerance to salinity varies for each species [9]. Salt-tolerant genes are responsible for diminishing the pace of the salt uptake and the transportation of the salt throughout the plant, as well as for adjusting the ionic and osmotic balance of the root and shoot cells and regulating leaf development [10].

Climate change is a factor that intensifies the salinization phenomenon, particularly in arid and semi-arid land (ASAL) regions and coastal areas, such as the Mediterranean basin [4]. The Mediterranean region will be affected by climate change as more extreme weather conditions, such as drought, temperature increases, and precipitation decreases, will occur [11]. Changes like rising sea levels will cause seawater intrusion resulting in irrigation problems, due to the high salinity levels [8]. Drought and low rainfall will cause salts to accumulate in soils, because of the water’s capillary rise and the upward movement of salts from the groundwater table to the surface [11]. Increased temperatures will result in higher evapotranspiration, which also increases salinity [1,4].

Research interest in wild non- or under-utilized species has grown, because of their high nutritional and medicinal value [12], their tolerance of abiotic stresses, such as salinity [13,14,15,16], and the ecosystem services they provide [17]. They are also vital for the protection of biodiversity, for sustainable development, and for economic resilience and seem to have a key role in dietary diversity and nutrition security [18,19]. *Sonchus oleraceus* L. is a wild edible leafy green of the Mediterranean basin, distributed worldwide, which is traditionally collected and commonly consumed after blanching or boiling in salads, pies, and soups [12,20]. The plant is recognized as a rich source of various nutrients and has promising medicinal properties [21,22]. Sonchus species exhibit notable adaptations to saline environments. More specifically, the seeds of *Sonchus oleraceus* and *Sonchus tenerrimus* have shown germination at high salinity levels and a tolerance of low–medium salinity stresses at the seedling stage [23,24], while *Sonchus arvensis* is reported to grow in saline environments [25,26,27].

To the best of the authors’ knowledge, there is limited information about the cultivation practices and needs for the commercial exploitation of *Sonchus oleraceus* L. [28,29,30] and a lack of published scientific literature concerning the plant’s tolerance of salinity during growth [31]. Considering all the factors mentioned above, this study hypothesized that *Sonchus oleraceus* L., as a wild species evolving under the environment’s abiotic stresses, could have been adapted to stress factors such as salinity. If so, it could be used in plant breeding as a source of salinity-resistant genes and introduced to the market as a new cultivation. Thus, this study aimed to evaluate the effects of varying salinity levels in the irrigation solution on the growth, quality, and nutrient composition of *Sonchus oleraceus* L. cultivation.

## 2. Results and Discussion

### 2.1. Growth Evaluation

Generally, salt tolerance is a complex characteristic mainly considered with respect to the maintenance of the yield and the growth rate. Other parameters such as plant survival, leaf size and number, root weight, water balance, quality, nutrient preservation, and the accumulation of specific ions without toxic effects in leaves and shoots are also used when measuring salt tolerance [32]. It is understood that salinity tolerance can be indicated by many growth attributes; however, the effects may vary between species, even in plants characterized as tolerant. The leaf and root growth parameters measured at the end of the experiment (D24) are shown in Table 1. In general, a clear linear regression was observed for salinity levels and yield (y = −3.3735x + 27.579, R^2^ = 0.9079), rosette diameter (y = −4.8583x + 46.525, R^2^ = 0.9742), and root fresh weight (y = −1.5527x + 10.302, R^2^ = 0.9352).

**Figure 1:**
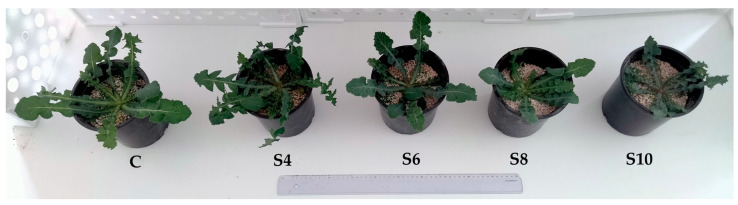
Size comparison at the end of the experiment of *Sonchus oleraceus* L. plants irrigated with nutrient solutions of different salinity levels [C: control (2.6 mS/cm), S4 (6.2 mS/cm), S6 (7.7 mS/cm), S8 (9.8 mS/cm), and S10 (11.4 mS/cm)].

The rosette diameter was significantly affected by high salinity levels (S8 and S10) showing a 38.3% and 46.8% reduction, respectively, compared to the control (C). Treatment S10 also had a significantly smaller rosette diameter compared to S4. At moderate salinity levels, the rosette diameter and, consequently, the leaf size were not affected. These results are also visualized in Figure 1. The same effects were found in a study on another Asteraceae wild leafy vegetable, *Reichardia picroides*, which showed that moderate salinity (6 mS/cm) did not significantly impact rosette diameter [14]. Compared to the C, the largest decrease in leaf fresh weight per plant was observed in treatments S8 and S10 (50.4% and 54.6% lower than the C, respectively), followed by S4 (a 27.5% decrease compared to the C). Treatment’s S6 leaf fresh weight was significantly higher than S8 and S10 and did not differ from the C and S4. The percentage of leaf dry matter in S6 was approximately 10% lower than the C and S8, with no other statistically significant differences observed between the treatments. In studies on relative species (*Taraxacum officinale*, *Reichardia picroides*, *Hedypnois cretica* L. and *Urospermum picroides* L.), the more tolerant species maintained leaf dry matter content at high salinity levels, but the leaf fresh weight was affected by both moderate and high salinity [14,16]. In the current experiment, salinity did not have an effect on leaf number at D0, D8, D16 (Figure S1), nor D24 (Table 1).

Similar to the leaves, chlorophyll fluorescence parameters Fv/Fm and PI abs, estimated at D0, D8, D16, and D24 (Figure S1), did not show any statistically significant difference between the treatments, within any day. Both parameters are used widely to determine the tolerance to environmental stresses, such as salinity, through the development of photosynthetically active plant organs, such as leaves [33,34]. Salinity tolerance in plants is determined by ion-independent (osmotic stress-related) effects, which have rapid and more intense consequences on plant growth [35], or ionic effects, which appear later in the plants’ development [36]. The delayed ionic effects involve either the exclusion of sodium (Na) from the leaves or tissue tolerance to accumulated Na or Cl (chlorine) [35]. Tolerance to osmotic stress is evident when there are no rapid reductions in turgor and stomatal closure, as well as in the rates of transpiration, photosynthesis, and growth [36]. Increased osmotic tolerance is indicated by the production of new leaves, while tissue tolerance is correlated with the survival of older leaves [35]. The results suggest that *Sonchus oleraceus* is tolerant to both osmotic and ionic stress, as there was no effect on the photosynthetic mechanism and the leaf production during the initial or final stages of the cultivation. This agrees with the literature that posits a close association between these two types of salt tolerance [35].

Electrolyte leakage (EL) was not affected by salinity. Another study, evaluating the impact of 0–300 mmol/L of NaCl on leafy vegetables (*Lactuca sativa* L., *Tetragonia tetragonoides* (Pall), and *Portulaca oleracea* L.), revealed a significant increase in the EL, with an increasing NaCl concentration for all species [37]. Generally, salinity, as a stress factor, increases the EL due to the increase in the membrane permeability [38]. In our study, the EL values remained constant among all treatments, which indicates an ability to tolerate excess salinity and maintain membrane stability and, consequently, leaf quality [39]. The RWC is an important index for the evaluation of the plant physiological status, which decreases in response to stressors such as salinity [40]. In the current experiment, although the RWC values decreased under saline conditions, no statistically significant difference was found, also suggesting a requirement for further investigation of the species’ tolerance to this kind of stress.

**Figure 2:**
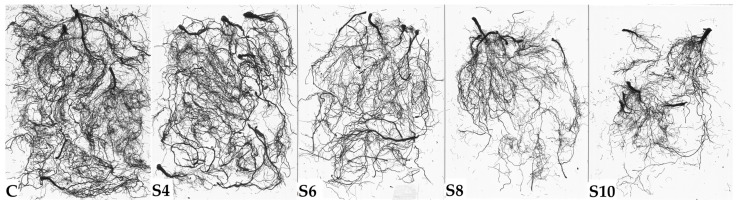
Root comparison at the end of the experiment of *Sonchus oleraceus* L. plants irrigated with nutrient solutions of different salinity levels [C: control (2.6 mS/cm), S4 (6.2 mS/cm), S6 (7.7 mS/cm), S8 (9.8 mS/cm), and S10 (11.4 mS/cm)] (one sample per picture).

At low salinity levels, root growth may be less affected, and sometimes even stimulated, compared to the shoot’s growth [32]. In our study, the root fresh weight was significantly decreased in high salinity levels (S8 and S10), showing a 62.3% and 68.2% reduction compared to the C. No statistically significant decrease in root fresh weight was found at moderate salinity levels (S4 and S6). Meanwhile, S10’s dry matter content was significantly higher than the C (45.9%), S4 and S6. Treatments S8 and S4 showed no significant differences in the root dry matter. Research on the rocket genotypes’ root fresh and dry weight indicated no salinity effects at 65 and 130 mM of NaCl [41]. The root surface area of the control plants was significantly higher than that of all treatments, with differences ranging from 46% to 64.9%, corresponding to increasing salinity levels. These results are visualized in Figure 2. However, salinity had no significant impact on the root tip number, although the root length was significantly affected in high salinity levels, indicating a 46.5% (S8) and a 46% (S10) decline compared to the C. In spinach, salinity significantly affected the root length at NaCl concentrations from 20 mM L^−1^ to 80 mM L^−1^ [42].

### 2.2. Quality Evaluation

Although salinity can either enhance the quality of vegetables or, especially on leafy vegetables, damage it [32,43], this was not the case in the current study. The quality was not affected, since no statistically significant differences were indicated between the treatments of any quality parameter analyzed (Table S1, Figure S1).

The ionic and osmotic stress caused by salinity can enhance the plants’ production of secondary metabolites, such as phenolic compounds and result in higher antioxidant activity [44]. The impact of saline irrigation water on three leafy vegetables of the Brassicaceae family grown hydroponically resulted in variable responses in the total phenolics content [45]. *Diplotaxis tenuifolia* and *Lepidium sativum* showed no significant differences among the five treatments (with an EC from 1.5 to 9.5 mS/cm) during the first trial. The same occurred in the second trial for *Lepidium sativum*; however, *Diplotaxis tenuifolia* showed an increase in total phenolic compounds in high salinity levels. *Eruca sativa* showed a reduction in total phenolic compounds under increased salinity during the first trial, but the opposite results occurred during the second trial. In our case, salinity had no statistically significant effect on *Sonchus oleraceus*’ total phenolics content and antioxidant activity. In research on green baby lettuce under similar conditions as in our study, antioxidant activity remained unaffected by salinity, as measured by the FRAP assay. However, phenolic content was observed to be higher in NaCl treatments [46]. *Sonchus oleraceus* is reported to have a high antioxidant capacity and phenolic content [47,48]; however, the total phenolics content and antioxidant activity values were relatively low but comparable with other wild leafy greens under salinity stress [16]. In the literature, the biochemical evaluation of *Sonchus oleraceus* is conducted from plants growing in the wild, where they are exposed to a variety of stress factors with no fertilization. It must be mentioned that phenolic compounds levels may vary depending on the species, physiological stage, and growth conditions [20].

The nitrate content in vegetables is a concerning factor because of its impact on human health [49]. As stated in the Official Journal of the European Union, Commission Regulation (EU) No 1258/2011, the European Commission has set limits on the maximum levels of nitrate content in foodstuffs, including green leafy vegetables, where it is most notably found [50]. There are no limits set by the EU specifically for nitrate content in Sonchus leaves. However, in the present study, the nitrate content was far below the maximum permissible values set for other leafy vegetables (lettuce, spinach, and rocket). Research on baby green and red lettuce in similar conditions as in the present study also found that the nitrate content was not affected by salinity, and the content was also lower than the threshold [46]. The literature on leafy vegetables suggests that a significant reduction in the accumulation of nitrate is possible under saline conditions [51]. Bian et al. state that the irrigation water quality should be seriously taken into account in vegetable production, especially under controlled environments, in order to achieve the production of low in nitrate vegetables [52].

Tissue tolerance to the accumulated Na is related to the biosynthesis of compatible solutes, such as sucrose [53]. Although the total soluble solids content was increased by salinity, no statistically significant differences were revealed in the present study. Thus, the salt tolerance of the plants is presumably ascribed to the synthesis of another solute, e.g., proline or glycine betaine. Similar results were observed in the relative species of wild leafy greens *Reichardia picroides* and *Taraxacum officinale* at 6 and 10 mS/cm [13]. A study on *Amaranthus gangeticus* found that the lightness and chroma color parameters were increased by salinity [54]. In our experiment, there were no statistically significant differences found between the treatments for the light parameters measured (lightness, chroma, and hue angle). Similarly, there was no significant effect of salinity on the chlorophyll content index between the treatments within any day examined (D0, D8, D16, and D24). Under salinity stress, the leaves of non-tolerant plants become yellow or brown due to the toxic effects of the Na accumulation, which leads to senescence and death [55]. The plants in this experiment not only survived even in high salinity, but also maintained their leaf quality without any evidence of color degradation or senescence, which also indicates their Na tolerance [35]. This result appears similar to the total chlorophyll content of pak choi (*Brassica rapa* ssp Chinensis L.) cultivation, which was not affected by salinity at EC levels of 9.5 and 12.3 mS/cm [56]. The total chlorophyll content of *Reichardia picroides* and *Taraxacum officinale* leaves was also not significantly affected by salinity at 6 and 10 mS/cm [14]. In four leafy cultivars of lettuce and pak choi, the relative chlorophyll content was not affected by low–mild salinity levels (3.20 mS/cm) in an NFT initial solution, except for the pak choi variety ‘Rosie’ [57].

### 2.3. Nutrient Composition

The composition of nitrogen (N), phosphorus (P), potassium (K), and sodium (Na) and the K/Na ratio determined from the leaf and root samples from D24 are shown in Table 2. Regarding the leaves, the N percentage was not affected by salinity, which agrees with previous studies on leafy vegetables [46,58]. The leaves’ P content was variably affected by salinity, indicating a statistically significant increase in treatments S4 and S8 compared to the C (32.1% and 42.6% increases, respectively). While S8 had the highest increase in the P content, S10 had the lowest among all the treatments compared to the C, which was 36.1% lower than S8. In the lettuce, the P percentage during a spring hydroponic cultivation was affected by salinity, in the same way as in the present experiment, i.e., at 40 mM of NaCl, there was a significant increase in the P percentage, while at 60 mM of NaCl there was no significant difference compared to the control [59].

The leaves’ K content was significantly affected by high salinity levels, showing a decrease of 20.8% in treatment S8 and 38.6% in treatment S10 compared to the C. Treatment S8 did not differ significantly from the treatments at moderate salinity levels, S4 and S6. The Na content in the leaves was the lowest in the C plants (8.57 ± 0.42 mg g^−1^ Leaf DW) and gradually increased with increasing NaCl concentrations. The ability of an optimal K/Na ratio regulation is a prerequisite of the salt stress tolerance [60,61], and the minimum value of the ratio in plant cells is approximately one [62]. The leaves’ K/Na ratio was significantly the highest in the C among all treatments. Moreover, the K/Na ratio remained close to the minimum value under moderate salinity and decreased further at high salinity levels, without showing any statistically significant difference. In moderate salinity, the plants were able to maintain their K concentration, but the K/Na ratio was affected by a high Na accumulation. In high salinity, low K/Na values were caused by the high content of Na in the tissues, in collaboration with the relatively low K content. Salinity tolerance is associated with the accumulation of excess Na in leaves and shoots, rather than uptake restriction [63]. As mentioned in Section 2.1, tolerance to salinity is closely associated with the management of osmotic and ionic effects. In this experiment, the Na accumulation in the leaves seems to have the main role in the plants’ tolerance to salinity. Ionic tolerance, specifically tolerance to Na, is observed in functional leaves, such as those of the plants in this experiment, due to the compartmentalization and sequestration of Na into the vacuoles of the cells [35]. Nevertheless, this hypothesis needs to be investigated for the case of *Sonchus oleraceus*. The WHO recommendation on daily Na intake is <2000 mg Na in adults, although the global average intake is estimated to be 4310 mg per day [64]. Thus, the health effects of the consumption of vegetables growing under salinity stress need to be researched thoroughly [65].

In the roots, there were no effects of salinity on the N, P, and K concentrations. The roots’ Na content was statistically significantly the lowest in the C among all the treatments. Similar to the leaves, the K/Na ratio was significantly the highest in the roots of treatment C, compared to all other treatments, and the lower ratio in saline conditions was undoubtedly a result of the higher Na concentrations in the root tissues. In some cases of wild leafy vegetables, the root K was decreased, while the P was increased, with increased salinity [14,16], but, generally, the root nutrient accumulation under salinity stress depends on the plant genotype and salinity source and level [66,67,68].

## 3. Materials and Methods

### 3.1. Experimental Setup

The experiment was conducted in an unheated plastic greenhouse at the Laboratory of Vegetable Crops, in the farm of Aristotle University of Thessaloniki, Greece (N 40.536; E 22.996) during March–April 2023. The *Sonchus oleraceus* L. seeds, provided by a seed company from Servia, Kozani, Greece (https://www.zouliamis.gr/, accessed on 13 March 2024), were sown in trays (cell dimensions 1 × 1 cm^2^; 14 plants per m^2^) filled with peat moss (Klassman-Deilmann KTS2). At a stage proximate to 6–7 leaves, 80 uniform seedlings were transplanted in 2 L pots with perlite as a growing medium. The experimental protocol was based on the comparison between nutrient solutions with no NaCl (the control) or containing 40 (S4), 60 (S6), 80 (S8), and 100 (S10) mM of NaCl. A completely randomized design (CRD) was used for the treatment distribution in the field. The temperature and relative humidity were monitored throughout the experiment, with an average temperature of 14 °C and a relative humidity of 60%.

### 3.2. Nutrient Solution

The composition of the nutrient solution used for irrigation, suitable for the needs of a wild leafy vegetable, was calculated using NUTRISENCE, an online Decision Support System (DSS) (https://nutrisense.online/, accessed on 23 February 2023): K^+^ 7.98 mmol/L, Ca^2+^ 4.70 mmol/L, Mg^2+^ 3.06 mmol/L, NH_4_^+^ 1.09 mmol/L, SO_4_^2−^ 4.99 mmol/L, NO_3_^−^ 12.93 mmol/L, H_2_PO_4_^−^ 1.40 mmol/L, Fe 20.00 μmol/L, Mn^++^ 9.00 μmol/L, Zn^++^ 5.00 μmol/L, Cu^++^ 0.80 μmol/L, B 30.00 μmol/L, Mo 0.50 μmol/L, Cl^−^ 0.64 mmol/L, Na^+^ 0.96 mmol/L, and HCO_3_^−^ 0.63 mmol/L. An EC of 2.6 mS/cm was set as the minimum value in order for the plants to be provided with every essential nutrient. Furthermore, the control plants (C) were irrigated with this nutrient solution [100% strength; a pH of 5.6; and an EC of 2.6 mS/cm], while treatments S4, S6, S8, and S10 were irrigated with the same nutrient solution as the C, with the addition of 40, 60, 80, and 100 mM of NaCl, respectively (100% strength; a pH of 5.6; and an EC of 6.2, 7.7, 9.8, and 11.4 mS/cm, respectively). Treatments S4 and S6 were categorized as “medium salinity” and treatments S8 and S10 as “high salinity”. Irrigation was performed every 2–3 days, depending on the pots’ drainage, with 15% of the nutrient solution draining off. The total volume of the irrigation applied during the experiment was 1.85 L per pot (approximately 135 mL per irrigation).

### 3.3. Growth Parameters

On the day of the transplantation (D0), as well as 8, 16, and 24 days after (D8, D16, and D24, respectively), the leaf number and the chlorophyll fluorescence parameters Fv/Fm (the maximum quantum yield of PSII) and PI abs (Performance Index) were measured with a chlorophyll fluorimeter (pocket PEA Chlorophyll Fluorimeter, Hansatech Instruments Ltd., Norfolk, UK). The Fv/Fm ratio provides information about the proportion of the light absorbed by the chlorophyll in PSII that is used in photochemical processes and PI abs is a suitable parameter to investigate the plants’ overall photosynthetic performance [33]. At the end of the experiment (D24), apart from the measurements mentioned above, the leaf and root fresh weight (FW), dry matter content (DMC), relative water content (RWC), and rosette diameter per plant were also determined. The RWC was evaluated as described by Smart and Bingham [69]. For the evaluation of the DMC, the leaves and roots were air-dried at 70 °C to a constant weight and estimated by dividing the DW by the FW, expressed in percentages. After the removal of any perlite left on the root samples, an image analysis of the roots took place (root scanner: EPSON Perfection V700, Nagano, Japan; and image analysis: WinRHIZO Pro, Regent Instruments Inc., Quebec City, QC, Canada) to determine the roots’ surface area (SA), tip number, and length.

### 3.4. Quality Traits Analyses

The relative chlorophyll content (chlorophyll content index, CCI) was estimated using a chlorophyll content meter (CCM-200, Opti-Sciences, Hudson, NH, USA) at D0, D8, D16, and D24. The leaf color from of fully developed leaves was measured at D24 with a chroma meter (Minolta CR-400, Osaka, Japan), and the colorimetric coordinates of lightness, hue angle, and chroma were evaluated. Lightness refers to the brightness; hue angle refers to the color in the form of a sphere where 0° is red, 90° is yellow,180° is green, and 270° is blue; and chroma refers to the color intensity [70].

The electrolyte leakage (EL) of the leaf samples from D24 was also determined, as described in the literature [37]. Additionally, leaf samples from D24 were homogenized and stored at −30 °C for phytochemical analyses. The total soluble solids (TSS) were evaluated with the use of a refractometer (Atago Co., Ltd., Tokyo, Japan). The total phenolics content (TPC) was determined by the Singleton and Rossi method, using gallic acid as a standard phenolic compound [71]. The phenolics were extracted with 80% aqueous methanol; 2.5 mL of 10% Folin–Ciocalteu reagent, 2 mL of 7.5% NaCO_3_, and 0.5 mL of the sample extract were incubated in a water bath for 5 min at 50 °C.The absorbance was measured at 760 nm using a spectrophotometer. The antioxidant capacity was measured using the Benzie and Strain ferric reducing antioxidant power (FRAP) assay [72]. The total antioxidants were extracted with 80% aqueous methanol; 0.1 µL of the sample extract and 3 mL of a solution consisting of CH_3_COONa (at a pH of 3.6), TPTZ, and FeCl_3_ were incubated in a water bath for 4 min at 37 °C.The absorbance was measured at 593 nm using a spectrophotometer. The nitrate content was evaluated according to the method of Cataldo et al. [73]. The samples were diluted in 25 mL of deionized water; 0.8 mL of 5% salicylic acid diluted in H_2_SO_4_ was added in a tube with 0.2 mL of the sample extract; 0.8 mL of H_2_SO_4_ was added in a second tube with 0.2 mL of the sample extract; and 19 mL of 2N NaOH was added to all the tubes. The absorbance was measured at 410 nm.

### 3.5. Nutrient Analyses

The leaf and root samples from D24 were collected for nutrient analyses. The samples were ashed at 500 °C for 6 h, and then dissolved in 2M HCl and filtered. The phosphorus (P), potassium (K), and sodium (Na) were determined in the filtrate. The P was determined calorimetrically according to the molybdenum blue–ascorbic acid method [74], while the K and Na were determined using a flame photometer. Lastly, the nitrogen (N) concentration was evaluated using the Kjeldahl method [75].

### 3.6. Statistical Analysis

The data were summarized by estimating the mean values and the corresponding Standard Errors (SE). All data (except for Leaf Number at D8 and D16, and PI abs at D0 and D8, as described below) were analyzed within the methodological frame of General Linear Models with the ANOVA method [76]. The models’ residuals were tested for normality and homoscedasticity. These assumptions were not met for Leaf FW, Root FW, RWC, Fv/Fm at D8 and D16, Leaf Number at D24, and PI abs at D24, and the corresponding data were log-transformed. The ANOVA method was applied to the log-transformed data. In the corresponding reported results, the mean values of the back-transformed data are presented. The mean values for the raw and transformed data were compared by the Tukey’s HSD test, except in the case of the Leaf K/Na where the mean values were compared through the Games–Howell post hoc test [77], since the corresponding data violated the homoscedasticity assumption, and the log transformation did not optimize this violation. In the case of the Leaf Number at D8 and D16 and the PI abs at D0 and D8, the log transformation did not optimize the models’ residuals’ normality and homoscedasticity, and the corresponding raw data were analyzed with the non-parametric ANOVA Kruskal–Wallis H test. Comparative analyses for Fv/Fm, PI abs, Leaf Number, and CCI, measured at D0, D8, D16, and D24, were conducted each day to evaluate the impact of salinity on each discrete stage of growth. In all the hypothesis testing procedures, the significance level was preset at a = 0.05 (*p* ≤ 0.05). The statistical analysis was performed using IBM SPSS Statistics v.28.0 software (SPSS 28.0, IBM Corp., Armonk, NY, USA).

## 4. Conclusions

To summarize, the effects of salinity in the irrigation of *Sonchus oleraceus* L. were investigated. In the leaves, the moderate salinity treatments (S4 and S6) each affected one out of eight parameters measured, while the high salinity treatments (both S8 and S10) affected two parameters. The root growth showed a higher reduction in four out of the five attributes measured in the S8 and S10 treatments, whereas treatments S4 and S6 affected only one parameter each. Hence, the salinity did not have a remarkable impact on the plant growth at a moderate salinity (S4 and S6). The leaf quality was stable across the salinity treatments. The N concentration was not affected by salinity, while the accumulation of P, K, and Na was variably affected by the salinity in the leaves but not in the roots, except in the case of Na. Due to the plant’s ability to tolerate the significant amounts of Na accumulated in the leaves, the effects on growth were minimal, particularly under moderate salinity, while no quality degradation could reduce the market value of the product.

In conclusion, *Sonchus oleraceus* L. showed a moderate tolerance to medium salinity stress, due to a high Na accumulation in leaves. Consequently, the exploitation of this species may hold value for its potential as an alternative cultivation in areas with poor-quality irrigation water. However, the accumulation of phytochemical compounds in plants grown under salinity stress must be studied. This study is a step towards further research on the effects of salinity on *Sonchus oleraceus* L. at a biochemical and molecular level. Lastly, since *Sonchus oleraceus* L. is a widespread species, further research on landraces’ distinctive characteristics of adaptation to each geographic region’s stressors could help gain a better understanding and utilization of this species.

## Figures and Tables

**Table 1:** Growth parameters at the end of the experiment (mean values ± SE) of *Sonchus oleraceus* L. plants irrigated with nutrient solutions of different salinity levels. Mean values within a row followed by a different letter(s) are statistically significantly different, at a significance level a = 0.05 (*p* ≤ 0.05), according to the results of ANOVA and Tukey’s HSD multiple comparisons procedure.

Growth Parameters	Treatments *				
	C	S4	S6	S8	S10
Rosette Diameter (cm) (n = 3)	42.92 ± 1.59 a	34.92 ± 3.36 ab	32.58 ± 3.96 abc	26.50 ± 1.46 bc	22.83 ± 0.96 c
Leaf FW ** (g) (n = 14)	25.62 ± 2.08 a	18.57 ± 1.71 b	18.84 ± 1.46 ab	12.70 ± 0.78 c	11.63 ± 0.83 c
Leaf DMC ** (%) (n = 3)	9.99 ± 0.20 a	9.57 ± 0.25 ab	8.98 ± 0.11 b	10.03 ± 0.14 a	9.72 ± 0.02 ab
Leaf Number (n = 6)	17 ± 1.45 a	18 ± 1.43 a	16 ± 2.49 a	13 ± 0.31 a	13 ± 0.56 a
Leaf RWC ** (%) (n = 3)	80.80 ± 1.14 a	76.17 ± 0.78 a	79.47 ± 3.19 a	76.27 ± 2.58 a	76.39 ± 0.69 a
EL ** (%) (n = 3)	19.59 ± 0.73 a	20.77 ± 0.94 a	21.52 ± 0.25 a	21.57 ± 1.08 a	24.34 ± 1.34 a
Root FW (g) (n = 14)	9.27 ± 0.39 a	6.37 ± 0.23 a	6.14 ± 1.45 a	3.50 ± 0.60 b	2.95 ± 1.07 b
Root DMC (%) (n = 3)	5.68 ± 0.37 c	5.96 ± 0.30 bc	5.65 ± 0.47 c	7.34 ± 0.11 ab	8.29 ± 0.35 a
Root SA ** (cm^2^) (n = 3)	682.86 ± 81.2 a	368.74 ± 52.9 b	321.39 ± 41.2 b	241.25 ± 11.7 b	239.44 ± 32.2 b
Root Tip Number (n = 3)	4747 ± 822 a	3777 ± 399 a	3025 ± 443 a	2706 ± 175 a	2884 ± 159 a
Root Length (m) (n = 3)	30.35 ± 3.75 a	22.40 ± 2.76 ab	19.84 ± 2.19 ab	16.25 ± 0.52 b	16.38 ± 2.20 b

* C: control (2.6 mS/cm), S4 (6.2 mS/cm), S6 (7.7 mS/cm), S8 (9.8 mS/cm), S10 (11.4 mS/cm), ** FW: Fresh Weight, DMC: Dry Matter Content, RWC: Relative Water Content, EL: Electrolyte Leakage, and SA: Surface Area.

**Table 2:** Leaf and root nutrient composition at the end of the experiment (mean values ± SE) of *Sonchus oleraceus* L. plants irrigated with a nutrient solution of different salinity levels. Mean values (n = 3) within a column (for the leaves and roots separately) followed by a different letter(s) are statistically significantly different, at a significance level a = 0.05 (*p* ≤ 0.05), according to the results of ANOVA and Tukey’s HSD multiple comparisons procedure (for all the parameters except for Leaf K/Na where the multiple comparisons were performed with the Games–Howell post hoc test).

	Treatment *	N	P	K	Na	K/Na
Leaves		(%)	(mg g^−1^ Leaf DW **)			-
	C	4.32 ± 0.18 a	2.91 ± 0.33 c	48.47 ± 1.66 a	8.57 ± 0.42 d	5.69 ± 0.40 a
	S4	4.12 ± 0.07 a	3.84 ± 0.03 ab	43.80 ± 1.30 ab	35.82 ± 2.17 c	1.24 ± 0.11 b
	S6	4.46 ± 0.21 a	3.68 ± 0.14 abc	40.33 ± 2.74 ab	42.28 ± 3.93 bc	0.98 ± 0.15 b
	S8	4.17 ± 0.16 a	4.15 ± 0.13 a	38.36 ± 1.79 bc	52.24 ± 1.69 ab	0.74 ± 0.06 b
	S10	4.19 ± 0.10 a	3.05 ± 0.14 bc	29.74 ± 1.96 c	62.12 ± 1.12 a	0.48 ± 0.03 b
**Roots**		**(%)**	**(mg g^−1^ Root DW)**			**-**
	C	3.80 ± 0.21 a	3.73 ± 0.21 a	23.84 ± 1.17 a	14.83 ± 1.2 b	1.63 ± 0.17 a
	S4	3.32 ± 0.02 a	3.30 ± 0.39 a	23.86 ± 1.19 a	31.14 ± 2.37 a	0.77 ± 0.06 b
	S6	3.43 ± 0.08 a	3.31 ± 0.26 a	28.47 ± 3.54 a	37.05 ± 1.68 a	0.78 ± 0.13 b
	S8	3.25 ± 0.17 a	3.92 ± 0.63 a	20.53 ± 2.37 a	40.11 ± 0.83 a	0.51 ± 0.06 b
	S10	3.23 ± 0.22 a	4.28 ± 0.70 a	21.76 ± 0.71 a	33.02 ± 3.82 a	0.67 ± 0.05 b

* C: control (2.6 mS/cm), S4 (6.2 mS/cm), S6 (7.7 mS/cm), S8 (9.8 mS/cm), and S10 (11.4 mS/cm); ** DW: Dry Weight.

## Data Availability

Data are contained within the article and Supplementary Materials.

## Supplementary Materials

The following supporting information can be downloaded at: https://www.mdpi.com/article/10.3390/plants13111552/s1, Figure S1: Mean values (n = 6) ± SE (see error bars) of chlorophyll fluorescence parameters Fv/Fm (A) and PI abs (B), Leaf Number (C), and Chlorophyll Content Index (CCI) (D) estimated on the first day of the experiment (D0), and 8, 16 and 24 days after (D8, D16 and D24, respectively); C: control (2.6 mS/cm), S4 (6.2 mS/cm), S6 (7.7 mS/cm), S8 (9.8 mS/cm), S10 (11.4 mS/cm). Mean values within a day followed by the same letter are not statistically significantly different, at significance level a = 0.05 (*p* ≤ 0.05), according to the results of ANOVA (for all the parameters except for Leaf Number at D8 and D16 and PI abs at D0 and D8 where the analysis was performed with the non-parametric ANOVA Kruskal-Wallis H test). Table S1: Quality traits (mean values ± SE) of *Sonchus oleraceus* L. plants irrigated with a nutrient solution of different salinity levels, at the end of the experiment. Mean values (n = 3) within a row followed by the same letter are not statistically significantly different, at significance level a=0.05 (*p* ≤ 0.05), according to the results of ANOVA.

## References

[1] Vengosh A. (2003). Salinization and Saline Environments. Treatise Geochem..

[2] Rasool S., Hameed A., Azooz M., Muneeb U., Siddiqi T., Ahmad P. (2013). Salt Stress: Causes, Types and Responses of Plants. Ecophysiology and Responses of Plants under Salt Stress.

[3] Hopmans J.W., Qureshi A.S., Kisekka I., Munns R., Grattan S.R., Rengasamy P., Ben-Gal A., Assouline S., Javaux M., Minhas P.S. (2021). Critical Knowledge Gaps and Research Priorities in Global Soil Salinity. Advances in Agronomy.

[4] Omuto C.T., Vargas R.R., El Mobarak A.M., Mohamed N., Viatkin K., Yigini Y. (2020). Mapping of Salt-Affected Soils—Technical Manual.

[5] Rhoades J.D., Kandiah A., Mashali A.M. (1992). The Use of Saline Waters for Crop Production.

[6] Giordano M., Petropoulos S.A., Rouphael Y. (2021). Response and Defence Mechanisms of Vegetable Crops against Drought, Heat and Salinity Stress. Agriculture.

[7] Zamann M., Shahidd S.A., Heng L. (2018). Guideline for Salinity Assessment, Mitigation and Adaptation Using Nuclear and Related Techniques.

[8] Mukhopadhyay R., Sarkar B., Jat H.S., Sharma P.C., Bolan N.S. (2021). Soil Salinity under Climate Change: Challenges for Sustainable Agriculture and Food Security. J. Environ. Manag..

[9] Minhas P.S., Ramos T.B., Ben-Gal A., Pereira L.S. (2020). Coping with Salinity in Irrigated Agriculture: Crop Evapotranspiration and Water Management Issues. Agric. Water Manag..

[10] Munns R. (2005). Genes and Salt Tolerance: Bringing Them Together. New Phytol..

[11] Corwin D.L. (2021). Climate Change Impacts on Soil Salinity in Agricultural Areas. Eur. J. Soil Sci..

[12] Panfili G., Niro S., Bufano A., D’Agostino A., Fratianni A., Paura B., Falasca L., Cinquanta L. (2020). Bioactive Compounds in Wild Asteraceae Edible Plants Consumed in the Mediterranean Diet. Plant Foods Hum. Nutr..

[13] Petropoulos S.A., Fernandes Â., Dias M.I., Pereira C., Calhelha R.C., Chrysargyris A., Tzortzakis N., Ivanov M., Sokovic M.D., Barros L. (2020). Chemical Composition and Plant Growth of *Centaurea raphanina* subsp. *mixta* Plants Cultivated under Saline Conditions. Molecules.

[14] Alexopoulos A.A., Assimakopoulou A., Panagopoulos P., Bakea M., Vidalis N., Karapanos I.C., Petropoulos S.A. (2021). Impact of Salinity on the Growth and Chemical Composition of Two Underutilized Wild Edible Greens: *Taraxacum officinale* and *Reichardia picroides*. Horticulturae.

[15] Calone R., Bregaglio S., Sanoubar R., Noli E., Lambertini C., Barbanti L. (2021). Physiological Adaptation to Water Salinity in Six Wild Halophytes Suitable for Mediterranean Agriculture. Plants.

[16] Alexopoulos A.A., Assimakopoulou A., Panagopoulos P., Bakea M., Vidalis N., Karapanos I.C., Rouphael Y., Petropoulos S.A. (2023). *Hedypnois cretica* L. and *Urospermum picroides* L. Plant Growth, Nutrient Status and Quality Characteristics under Salinity Stress. Horticulturae.

[17] Platis D.P., Papoui E., Bantis F., Katsiotis A., Koukounaras A., Mamolos A.P., Mattas K. (2023). Underutilized Vegetable Crops in the Mediterranean Region: A Literature Review of Their Requirements and the Ecosystem Services Provided. Sustainability.

[18] Mattas K., Raptou E., Alayidi A., Yener G., Baourakis G. (2023). Assessing the Interlinkage between Biodiversity and Diet through the Mediterranean Diet Case. Adv. Nutr..

[19] Mattas K., Nastis S.A., Michailidis A., Tsakiridou E., Koutroubas S. (2024). Unveiling the Hidden Gems: Minor Crops as Catalysts for Sustainable Development, Biodiversity Conservation, and Economic Resilience. Sustain. Dev..

[20] Disciglio G., Tarantino A., Frabboni L., Gagliardi A., Giuliani M.M., Tarantino E., Gatta G. (2017). Qualitative Characterisation of Cultivated and Wild Edible Plants: Mineral Elements, Phenols Content and Antioxidant Capacity. Ital. J. Agron..

[21] Guil-Guerrero J.L., Giménez-Giménez A., Rodríguez-García I., Torija-Isasa M.E. (1998). Nutritional Composition of Sonchus Species (*S asper* L., *S oleraceus* L. and *S tenerrimus* L.). J. Sci. Food Agric..

[22] Li X.M., Yang P.L. (2018). Research Progress of Sonchus Species. Int. J. Food Prop..

[23] Chauhan B., Gill G., Preston C. (2009). Factors Affecting Seed Germination of Annual Sowthistle (*Sonchus oleraceus*) in Southern Australia. Weed Sci..

[24] Khan Z., Albrecht M., Traveset A. (2013). Salt Application as an Effective Measure to Control Ruderal Invaders Threatening Endangered Halophytic Plant Species. Appl. Veg. Sci..

[25] Johnson-Green P.C., Kenkel N.C., Booth T. (1995). The Distribution and Phenology of Arbuscular Mycorrhizae along an Inland Salinity Gradient. Can. J. Bot..

[26] Naz I., Bano A., Hassan T. (2009). Isolation of Phytohormones Producing Plant Growth Promoting Rhizobacteria from Weeds Growing in Khewra Salt Range, Pakistan and Their Implication in Providing Salt Tolerance to *Glycine max* L.. Afr. J. Biotechnol..

[27] Zhao Y., Li X.-R., Zhang Z.-S., Hu Y.-G., Wu P. (2014). Soil-Plant Relationships in the Hetao Irrigation Region Drainage Ditch Banks, Northern China. Arid Land Res. Manag..

[28] Carrascosa Á., Pascual J.A., Ros M., Petropoulos S., del Mar Alguacil M. (2022). The Effect of Fertilization Regime on Growth Parameters of *Sonchus oleraceus* and Two Genotypes of *Portulaca oleracea*. Biol. Life Sci. Forum.

[29] Chrysargyris A., Goumenos C., Tzortzakis N. (2023). Use of Medicinal and Aromatic Plant Residues for Partial Peat Substitution in Growing Media for *Sonchus oleraceus* Production. Agronomy.

[30] Chrysargyris A., Tzortzakis N. (2023). Optimising Fertigation of Hydroponically Grown Sowthistle (*Sonchus oleraceus* L.): The Impact of the Nitrogen Source and Supply Concentration. Agric. Water Manag..

[31] Salonikioti A., Petropoulos S., Antoniadis V., Levizou E., Alexopoulos A. (2015). Wild Edible Species with Phytoremediation Properties. Procedia Environ. Sci..

[32] Shannon M.C., Grieve C.M. (1998). Tolerance of Vegetable Crops to Salinity. Sci. Hortic..

[33] Lepeduš H., Brkić I., Cesar V., Jurković V., Antunović Dunić J., Jambrović A., Brkić J., Šimić D. (2012). Chlorophyll Fluorescence Analysis of Photosynthetic Performance in Seven Maize Inbred Lines under Water-Limited Conditions. Period Biol..

[34] Tao R., Ding J., Li C., Zhu X., Guo W., Zhu M. (2021). Evaluating and Screening of Agro-Physiological Indices for Salinity Stress Tolerance in Wheat at the Seedling Stage. Front. Plant Sci..

[35] Munns R., Tester M. (2008). Mechanisms of Salinity Tolerance. Annu. Rev. Plant Biol..

[36] Morton M.J.L., Awlia M., Al-Tamimi N., Saade S., Pailles Y., Negrão S., Tester M. (2019). Salt Stress under the Scalpel—Dissecting the Genetics of Salt Tolerance. Plant J..

[37] Hniličková H., Hnilička F., Orsák M., Hejnák V. (2019). Effect of Salt Stress on Growth, Electrolyte Leakage, Na+ and Κ+ Content in Selected Plant Species. Plant Soil Environ..

[38] Dhindsa R.S., Plumbdhindsa P., Thorpe T.A. (1981). Leaf Senescence: Correlated with Increased Levels of Membrane Permeability and Lipid Peroxidation, and Decreased Levels of Superoxide Dismutase and Catalase. J. Exp. Bot..

[39] Romero-Romero J.L., Inostroza-Blancheteau C., Reyes-Díaz M., Matte J.P., Aquea F., Espinoza C., Gil P.M., Arce-Johnson P. (2020). Increased Drought and Salinity Tolerance in *Citrus aurantifolia* (Mexican Lemon) Plants Overexpressing Arabidopsis CBF3 Gene. J. Soil Sci. Plant Nutr..

[40] Abdelaal K., Alsubeie M.S., Hafez Y., Emeran A., Moghanm F., Okasha S., Omara R., Basahi M.A., Darwish D.B.E., Ibrahim M.F.M. (2022). Physiological and Biochemical Changes in Vegetable and Field Crops under Drought, Salinity and Weeds Stresses: Control Strategies and Management. Agriculture.

[41] Petretto G.L., Urgeghe P.P., Massa D., Melito S. (2019). Effect of Salinity (NaCl) on Plant Growth, Nutrient Content, and Glucosinolate Hydrolysis Products Trends in Rocket Genotypes. Plant Physiol. Biochem..

[42] Abbas A., Siddiq Z., Hayyat M.U., Zhang Y.J., Ghaffar R., Gatasheh M.K. (2024). Na+ and K+ Compartmentalization in *Spinacea oleracea* and Their Effects on Growth, Water Relations, Endogenous Melatonin, and Non-Structural Carbohydrates. Sci. Hortic..

[43] Rouphael Y., Petropoulos S.A., Cardarelli M., Colla G. (2018). Salinity as Eustressor for Enhancing Quality of Vegetables. Sci. Hortic..

[44] Ramakrishna A., Ravishankar G.A. (2011). Influence of Abiotic Stress Signals on Secondary Metabolites in Plants. Plant Signal Behav..

[45] Hamilton J.M., Fonseca J.M. (2010). Effect of Saline Irrigation Water on Antioxidants in Three Hydroponically Grown Leafy Vegetables: *Diplotaxis tenuifolia*, *Eruca sativa*, and *Lepidium sativum*. HortScience.

[46] Neocleous D., Koukounaras A., Siomos A.S., Vasilakakis M. (2014). Assessing the Salinity Effects on Mineral Composition and Nutritional Quality of Green and Red “Baby” Lettuce. J. Food Qual..

[47] McDowell A., Thompson S., Stark M., Ou Z.Q., Gould K.S. (2011). Antioxidant Activity of Puha (*Sonchus oleraceus* L.) as Assessed by the Cellular Antioxidant Activity (CAA) Assay. Phytother. Res..

[48] Aissani F., Grara N., Bensouici C., Bousbia A., Ayed H., Idris M.H.M., Teh L.K. (2022). Algerian *Sonchus oleraceus* L.: A Comparison of Different Extraction Solvent on Phytochemical Composition, Antioxidant Properties and Anti-Cholinesterase Activity. Adv. Tradit. Med..

[49] Bryan N.S., van Grinsven H. (2013). The Role of Nitrate in Human Health. Advances in Agronomy.

[50] The European Commission (2011). Commission Regulation (EU) No 1258/2011 of 2 December 2011 amending Regulation (EC) No 1881/2006 as regards maximum levels for nitrates in foodstuffs. Off. J. Eur. Union.

[51] Rouphael Y., Kyriacou M.C. (2018). Enhancing Quality of Fresh Vegetables through Salinity Eustress and Biofortification Applications Facilitated by Soilless Cultivation. Front. Plant Sci..

[52] Bian Z., Wang Y., Zhang X., Li T., Grundy S., Yang Q., Cheng R. (2020). A Review of Environment Effects on Nitrate Accumulation in Leafy Vegetables Grown in Controlled Environments. Foods.

[53] Roy S.J., Negrão S., Tester M. (2014). Salt Resistant Crop Plants. Curr. Opin. Biotechnol..

[54] Sarker U., Hossain M.N., Oba S., Ercisli S., Marc R.A., Golokhvast K.S. (2023). Salinity Stress Ameliorates Pigments, Minerals, Polyphenolic Profiles, and Antiradical Capacity in Lalshak. Antioxidants.

[55] Negrão S., Schmöckel S.M., Tester M. (2017). Evaluating Physiological Responses of Plants to Salinity Stress. Ann. Bot..

[56] Mahmud T.M.M., Atherton J.G., Wright C.J., Ramlan M.F., Ahmad S.H. (1999). Pak Choi (*Brassica rapa* ssp *Chinensis* L.) Quality Response to Pre-Harvest Salinity and Temperature. J. Sci. Food Agric..

[57] Niu G., Sun Y., Masabni J.G. (2018). Impact of Low and Moderate Salinity Water on Plant Performance of Leafy Vegetables in a Recirculating NFT System. Horticulturae.

[58] Voutsinos-Frantzis O., Karavidas I., Petropoulos D., Zioviris G., Fortis D., Ntanasi T., Ropokis A., Karkanis A., Sabatino L., Savvas D. (2023). Effects of NaCl and CaCl_2_ as Eustress Factors on Growth, Yield, and Mineral Composition of Hydroponically Grown *Valerianella locusta*. Plants.

[59] Breś W., Kleiber T., Markiewicz B., Mieloszyk E., Mieloch M. (2022). The Effect of NaCl Stress on the Response of Lettuce (*Lactuca sativa* L.). Agronomy.

[60] Maathuis F.J.M., Sanders D. (1997). Regulation of K+ Absorption in Plant Root Cells by External K+: Interplay of Different Plasma Membrane K+ Transporters. J. Exp. Bot..

[61] Asch F., Dingkuhn M., Dörffling K., Miezan K. (2000). Leaf K/Na Ratio Predicts Salinity Induced Yield Loss in Irrigated Rice. Euphytica.

[62] Maathuis F.J.M., Amtmann A. (1999). K+ Nutrition and Na+ Toxicity: The Basis of Cellular K+/Na+ Ratios. Ann. Bot..

[63] Zurayk R.A., Baalbaki R. (1996). *Inula crithmoides*: A Candidate Plant for Saline Agriculture. Arid Soil Res. Rehabil..

[64] FAO (2022). Global Symposium on Salt-Affected Soils: Outcome Document.

[65] Das Shuvo S., Zahid A., Rahman M.M., Parvin R. (2020). Exploring the Impact of Soil and Water Salinity on Dietary Behavior and Health Risk of Coastal Communities in Bangladesh. J. Water Health.

[66] Loupassaki M.H., Chartzoulakis K.S., Digalaki N.B., Androulakis I.I. (2002). Effects of Salt Stress on Concentration of Nitrogen, Phosphorus, Potassium, Calcium, Magnesium, and Sodium in Leaves, Shoots, and Roots of Six Olive Cultivars. J. Plant Nutr..

[67] Fisarakis I., Nikolaou N., Tsikalas P., Therios I., Stavrakas D. (2005). Effect of Salinity and Rootstock on Concentration of Potassium, Calcium, Magnesium, Phosphorus, and Nitrate–Nitrogen in Thompson Seedless Grapevine. J. Plant Nutr..

[68] Ntatsi G., Aliferis K.A., Rouphael Y., Napolitano F., Makris K., Kalala G., Katopodis G., Savvas D. (2017). Salinity Source Alters Mineral Composition and Metabolism of *Cichorium spinosum*. Environ. Exp. Bot..

[69] Smart R.E., Bingham G.E. (1974). Rapid Estimates of Relative Water Content. Plant Physiol..

[70] Mcguire R.G. (1992). Reporting of Objective Color Measurements. HortScience.

[71] Singleton V.L., Joseph A.R. (1965). Colorimetry of Total Phenolics with Phosphomolybdic-Phosphotungstic Acid Reagents. Am. J. Enol. Vitic..

[72] Jones A., Pravadali-Cekic S., Dennis G.R., Bashir R., Mahon P.J., Shalliker R.A. (2017). Ferric Reducing Antioxidant Potential (FRAP) of Antioxidants Using Reaction Flow Chromatography. Anal. Chim. Acta.

[73] Cataldo D., Maroon M., Schrader L., Youngs V. (1975). Rapid Colorimetric Determination of Nitrate in Plant-Tissue by Nitration of Salicylic-Acid. Commun. Soil Sci. Plant Anal..

[74] Kuo S., Sparks D.L. (1996). Phosphorus. Methods of Soil Analysis—Part 3—Chemical Methods.

[75] Kjeldahl J. (1883). A New Method for the Determination of Nitrogen in Organic Matter. J. Anal. Chem..

[76] Steel R.G.D., Torrie J.H., Dickey D.A. (1997). Principles and Procedures of Statistics: A Biometrical Approach.

[77] Toothaker E.L. (1993). Multiple Comparison Procedures.

