# Supplementary material for: The Impact of Salinity in the Irrigation of a Wild Underutilized Leafy Vegetable, Sonchus oleraceus L
Source: Plants (Basel). 2024 Jun 4;13(11):1552. doi: 10.3390/plants13111552 (PMC11174866; doi:10.3390/plants13111552)
Supplement: Supplementary file 1 [file plants-13-01552-s001.zip › plants-2939514-supplementary.pdf]

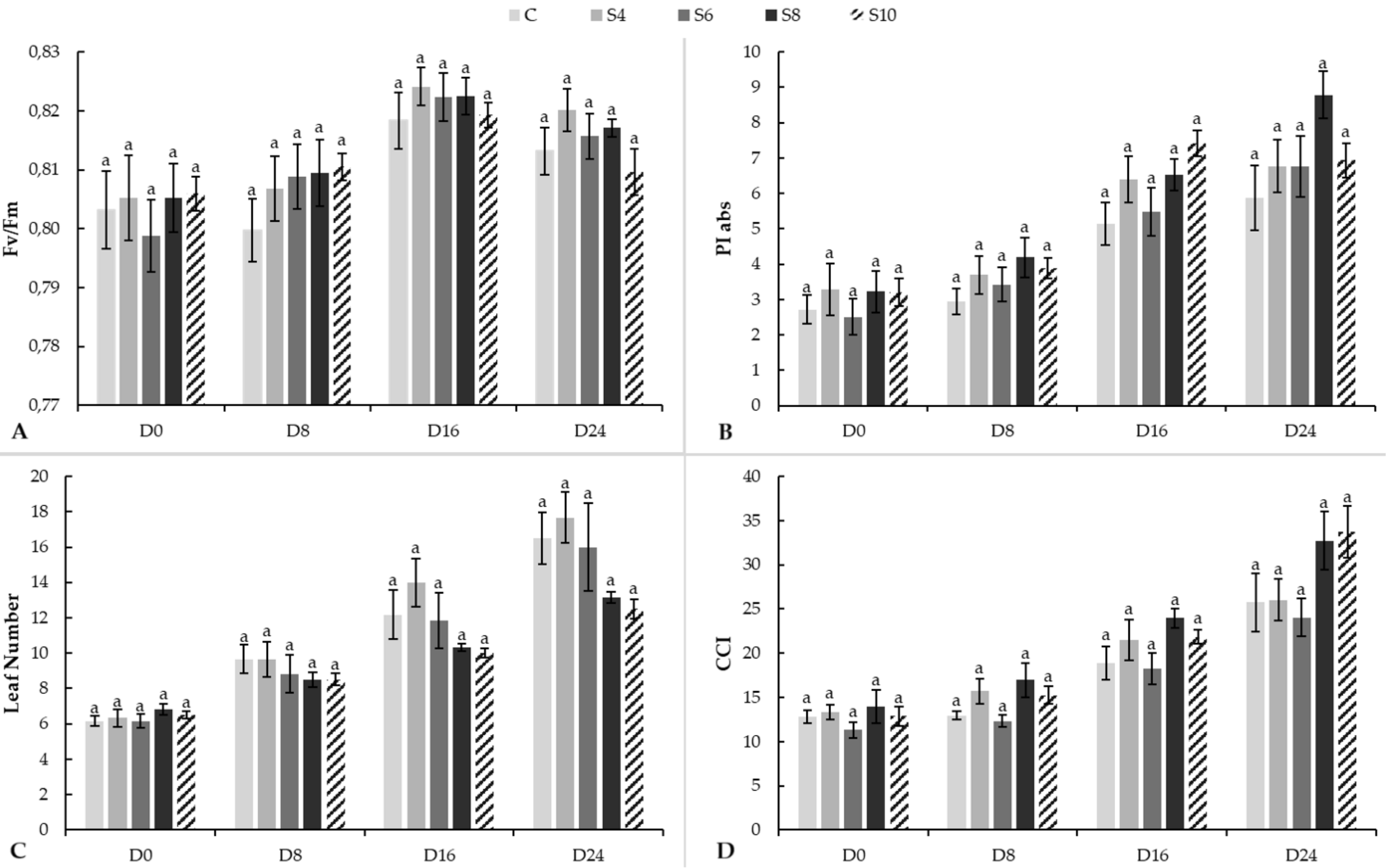

Figure S1. Mean values (n = 6) ± SE (see error bars) of chlorophyll fluorescence parameters Fv/Fm (A) and PI abs (B), Leaf Number (C), and Chlorophyll Content Index (CCI) (D) estimated on the first day of the experiment (D0), and 8, 16 and 24 days after (D8, D16 and D24, respectively); C: control (2.6 mS/cm), S4 (6.2 mS/cm), S6 (7.7 mS/cm), S8 (9.8 mS/cm), S10 (11.4 mS/cm). Mean values within a day followed by the same letter are not statistically significantly different, at significance level a=0.05 ($p \leq 0.05$), according to the results of ANOVA (for all the parameters except for Leaf Number at D8 and D16 and PI abs at D0 and D8 where the analysis was performed with the non-parametric ANOVA Kruskal-Wallis H test).

Table S1. Quality traits (mean values ± SE) of *Sonchus oleraceus* L. plants irrigated with a nutrient solution of different salinity levels, at the end of the experiment. Mean values (n = 3) within a row followed by the same letter are not statistically significantly different, at significance level a=0.05 ($p \leq 0.05$), according to the results of ANOVA.

Quality Traits	Treatments*				
	C	S4	S6	S8	S10
TSS** (°Brix)	6.57 ± 0.43 a	7.60 ± 0.21 a	7.30 ± 0.15 a	8.00 ± 0.25 a	8.23 ± 0.69 a
TPC** (mg g^{-1} FW**)	0.58 ± 0.08 a	0.45 ± 0.05 a	0.44 ± 0.07 a	0.50 ± 0.02 a	0.59 ± 0.09 a
FRAP** (mg g^{-1} FW)	262.3 ± 42.1 a	186.78 ± 13.7 a	206.02 ± 24.4 a	261.68 ± 13.8 a	248.05 ± 40.3 a
Nitrate (μg g^{-1} FW)	675.36 ± 51.5 a	608.66 ± 87.2 a	594.53 ± 28.2 a	703.55 ± 103.3 a	624.42 ± 102.4 a
Chroma	15.83 ± 0.88 a	16.10 ± 1.12 a	12.06 ± 0.47 a	14.05 ± 0.97 a	13.94 ± 1.04 a
Lightness	34.85 ± 0.56 a	35.40 ± 1.20 a	35.67 ± 1.20 a	36.49 ± 1.19 a	36.83 ± 1.71 a
Hue	132.89 ± 0.7 a	132.03 ± 1.0 a	132.36 ± 0.9 a	133.03 ± 0.9 a	132.41 ± 0.5 a

*C: control (2.6 mS/cm), S4 (6.2 mS/cm), S6 (7.7 mS/cm), S8 (9.8 mS/cm), S10 (11.4 mS/cm), ** TSS: Total Soluble Solids, TPC: Total Phenolic Compounds, FW: Fresh Weight, FRAP: Ferric Reducing Antioxidant Power (antioxidant compounds).
